# Adapting myoelectric control in real-time using a virtual environment

**DOI:** 10.1186/s12984-019-0480-5

**Published:** 2019-01-16

**Authors:** Richard B. Woodward, Levi J. Hargrove

**Affiliations:** 1 Center for Bionic Medicine, Shirley Ryan Ability Lab, Chicago, IL 60611 USA; 2 Department of Physical Medicine & Rehabilitation, Northwestern University, Chicago, IL 60611 USA; 3 Department of Biomedical Engineering, Northwestern University, Evanston, IL 60208 USA

**Keywords:** Amputee, Electromyography, Upper-limb prostheses, Pattern recognition, Virtual rehabilitation, Virtual guided training, Serious gaming, Real-time adaptation, Myoelectric control

## Abstract

**Background:**

Pattern recognition technology allows for more intuitive control of myoelectric prostheses. However, the need to collect electromyographic data to initially train the pattern recognition system, and to re-train it during prosthesis use, adds complexity that can make using such a system difficult. Although experienced clinicians may be able to guide users to ensure successful data collection methods, they may not always be available when a user needs to (re)train their device.

**Methods:**

Here we present an engaging and interactive virtual reality environment for optimal training of a myoelectric controller. Using this tool, we evaluated the importance of training a classifier actively (i.e., moving the residual limb during data collection) compared to passively (i.e., maintaining the limb in a single, neutral orientation), and whether computational adaptation through serious gaming can improve performance.

**Results:**

We found that actively trained classifiers performed significantly better than passively trained classifiers for non-amputees (*P* < 0.05). Furthermore, collecting data passively with minimal instruction, paired with computational adaptation in a virtual environment, significantly improved real-time performance of myoelectric controllers.

**Conclusion:**

These results further support previous work which suggested active movements during data collection can improve pattern recognition systems. Furthermore, adaptation within a virtual guided serious game environment can improve real-time performance of myoelectric controllers.

**Electronic supplementary material:**

The online version of this article (10.1186/s12984-019-0480-5) contains supplementary material, which is available to authorized users.

## Background

In the US, more than 600,000 people are estimated to be living with an upper limb amputation as a result of trauma, dysvascular disease, or cancer [1]. Although prostheses have been in use for centuries [2], they still lack the functionality and dexterity of a human hand/arm, resulting in device abandonment and diminished functional outcomes [3, 4].

Myoelectric devices—which are controlled by electromyographic (EMG) signals generated by contraction of residual muscles—provide many benefits over body-powered prostheses. Along with providing more degrees of freedom (DOFs) and the addition of net power to assist in grasping heavy items, myoelectric devices are typically more intuitive to control. The user can control the prosthesis by contracting muscles that would be used to perform desired postures (e.g., hand open, wrist flexion) in an intact limb [5], in contrast to body-powered devices, where shoulder movements are used to control the hand.

Pattern recognition (PR) techniques can be used to control myoelectric devices. Typically, EMG activity information recorded from the residual limb is used to train an algorithm to recognise which muscles are contracting and at what level during a given movement (e.g. hand open). Once trained, the PR system monitors the user’s muscle contractions and uses this information to estimate what movement the user is attempting to perform.

Although PR may provide improved control of myoelectric devices, it has some disadvantages. Typically, PR users collect EMG signals from each hand/wrist posture with the arm in one position—either by their side or parallel with the ground [6–8]. Although this passive data collection approach allows high classification rates in offline analysis, it does not necessarily translate well to real-time performance [9]. Previous studies have shown that active data collection—i.e., moving the arm during data collection, or simply collecting multiple sets of data for each posture in a variety of arm orientations, may improve real-time control [10–14]. Teaching end-users or inexperienced clinicians the subtleties of how to properly collect data to train a PR control system can be complex and time consuming. Clinicians and engineers with experience and a priori knowledge of PR control systems can readily identify poor habits and mistakes in data collection, providing users the best chance of reliable control of their prosthesis. However, involving these professionals is expensive, and they are not always available when training needs to be performed.

Past research has shown the benefits of online computational adaptation—the process of improving classification rates by incorporating additional data in real-time—for overall better performance [15–17]. Likewise, virtual rehabilitation and serious gaming have been used in recent years as alternative methods to facilitate myoelectric training [18–23]. The work introduced here combines these two research areas.

We present a tool for guiding the training of a PR system for myoelectric prosthesis control, using a virtual reality (VR) interface to create an engaging, interactive environment. We evaluate the importance of actively training a classifier (i.e., with the arm in different positions) versus the traditional method of passive training (with the limb held in a single neutral position) and show that serious gaming and computational adaptation of a classifier can improve myoelectric control in real-time.

## Methods

Sixteen individuals with intact limbs (ITL: eight male, eight female, aged between 22 and 35, with forearm circumferences between 21 and 29 cm), and four subjects with major upper limb amputations (AMP: three male, one female, aged between 31 and 69, with forearm circumferences between 20 and 27 cm, all with traumatic amputations occurring between four and 45 years ago, three transradial level, one wrist disarticulation level) were recruited for this study. AMP individuals had varying experience with myoelectric control and PR, ranging from use within a research environment to daily use of a PR-controlled myoelectric system, including the Complete Control system (Coapt LLC). Thus all AMP individuals could be considered at least intermediate users of the technology used in this study.

Half of the ITL subjects (*n* = 8) were randomly placed in an ‘adaptation group’, where new data were applied to the existing classifier to improve posture estimation, while the remaining subjects (*n* = 8) were placed in a ‘fixed group’, where performance was not computationally adapted. Subjects were not told which group they were in until after the study, and both groups performed the same study. For the AMP population, all participants (*n* = 4) were placed in a separate adaptation group, and there was no fixed group. ITL individuals had a broader range of myoelectric PR experience then the AMP group, with some with extensive experience and others with no experience at all.

### Hardware and signal processing

**Fig. 1:**
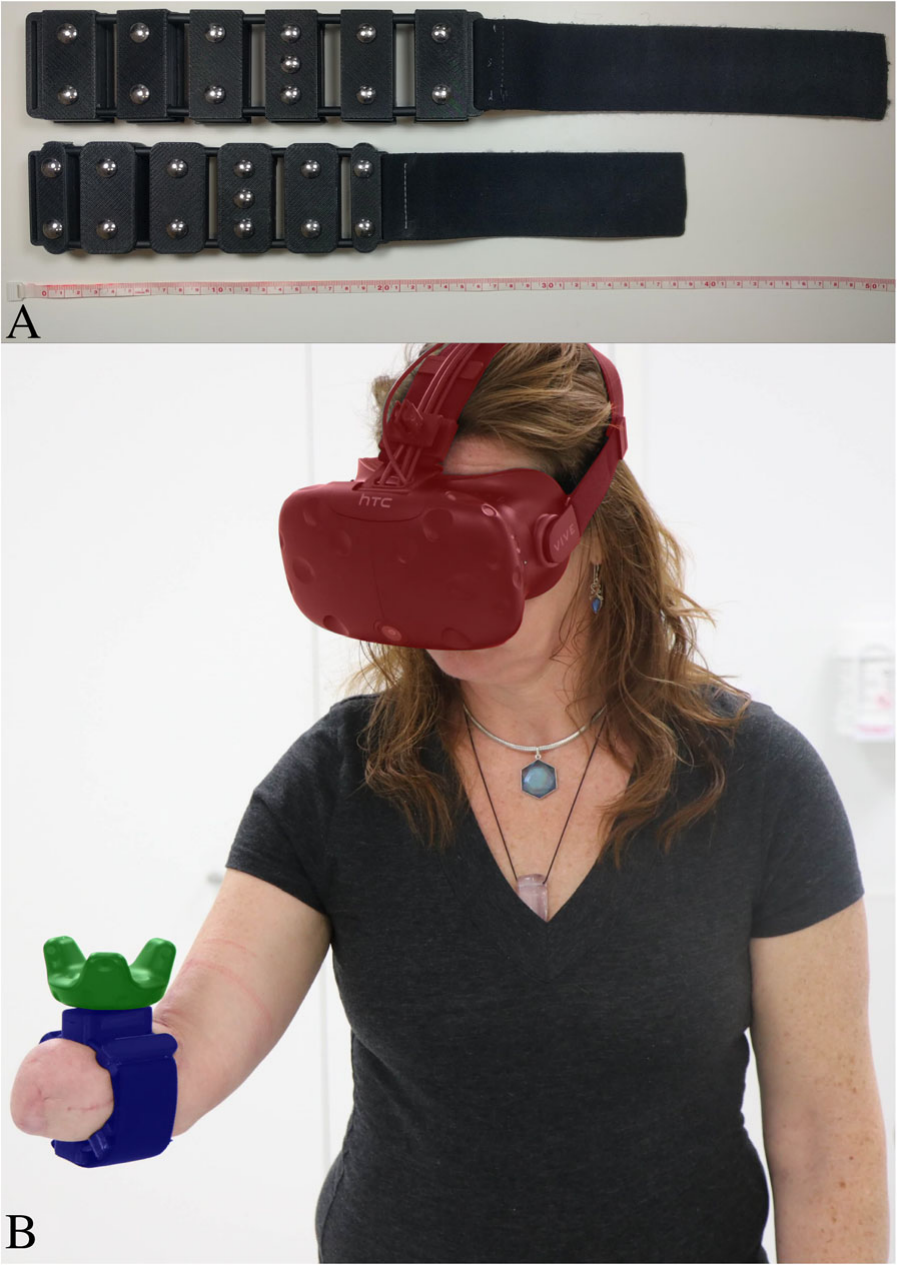
Technology used in Study. **a** Side-by-side view of the armbands used in this study, showing dome electrodes. Ruler (cm) used for scale; **b** Participant interacting in the virtual environment. The EMG embedded armband (blue) was wrapped around the subject’s upper forearm, while the VR tracker (green) and VR headset (red) tracked the subject’s movements and displayed the virtual environment

Two custom-fabricated EMG acquisition armbands (a small and a large size) were created to collect EMG data, perform PR on the collected data, and track movement of the arm/residual limb (Fig. 1a). The armband included six pairs of stainless steel dome electrodes (Motion Control Inc.) with inter-electrode spacings of approximately 2.5 cm. Signals were amplified using a TI ADS1299 bioinstrumentation chip programmed with a hardware gain of eight, sampled at 1000 Hz, and digitally filtered with a passband between 35 and 350 Hz. PR was performed using an embedded system on module (Logic PD SOMDM3730). Data were binned into 200 ms windows with a 175 ms overlap, resulting with a classifier estimation decision every 25 ms. Window size and overlap were selected based on previous work that recommended an optimal window length of between 150 and 250 ms [24], and a delay of no more than 100 ms [25]. Mean relative value, waveform vertical length, zero crossing, slope change, and auto-regressive features from a 6th order model were extracted from the data [26, 27], and classified with a linear discriminant analysis (LDA) algorithm. An estimate of movement velocity was constructed using an advanced proportional control algorithm as described by Scheme et al. [28], and the speed was smoothed using a velocity smoothing ramp as described by Simon et al. [29]. Classification and proportional control speed were processed on the embedded system and transmitted over Wi-Fi to a desktop computer (Alienware Aurora R5, Intel i7–6700, 16 GB RAM, NVIDIA GTX 1080). The data were then used to control objects in a virtual environment, which was developed in Unity (Version 2017.1.0f3). The VR system (HTC Vive) comprised a headset and tracking puck, both worn by the subject. The headset tracked participants’ head movements and displayed the virtual environment. The tracking puck was attached to the EMG armband and tracked subjects’ arm movements (Fig. 1b).

### Experimental tasks

The armband size that best fit the user was donned circumferentially around the forearm, just distal to the elbow. Hand/wrist postures used to train the classifier included no motion, hand open, hand close, wrist pronation, wrist supination, wrist flexion, and wrist extension. Both ITL and AMP subjects performed the same protocol, except that AMP subjects did not train or perform the wrist flexion/extension postures. AMP individuals typically use their affected muscles less following amputation, resulting in weaker strength [30]. To minimise fatigue, which may occur faster than in ITL individuals, the flexion/extension postures were removed in the AMP group. The five postures correspond to the functionality available in commercial prostheses, as no commercial flexor/extensor device currently exists. The experiment was split into three sections: initial data collection and training, real-time testing of control, and the gaming environment, with or without adaptation.

### Data collection and training

**Fig. 2:**
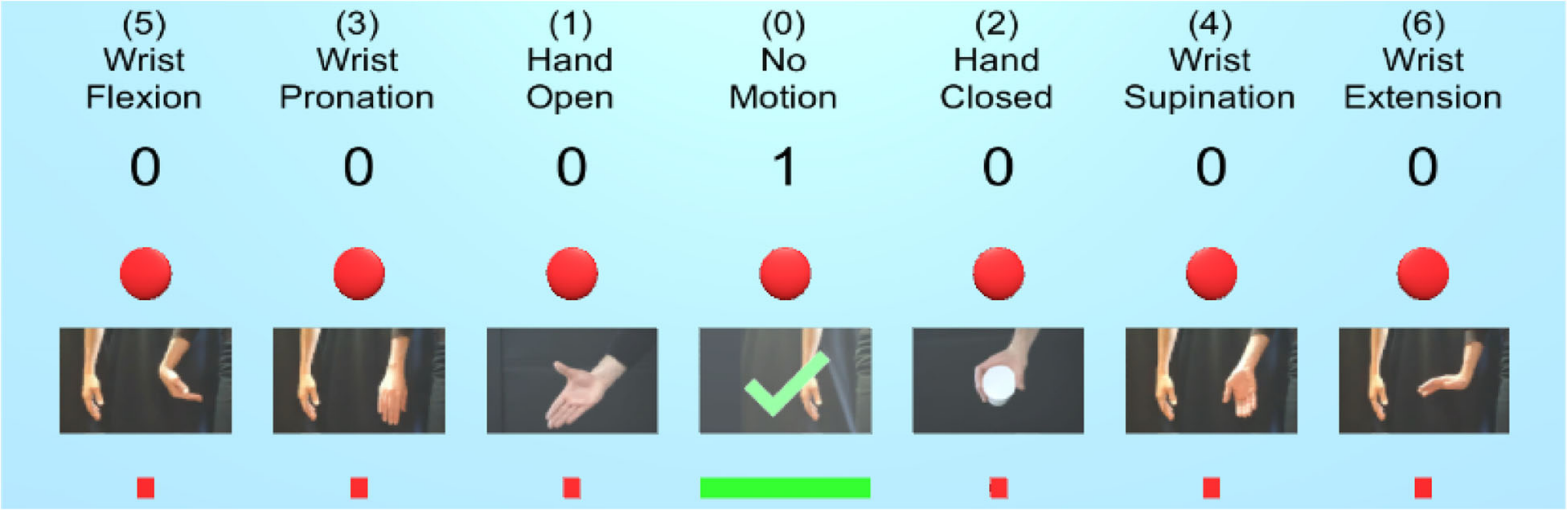
The pattern recognition collection ‘wall’ provided the user with feedback on their data collection progress. The titles and images indicated which postures they needed to perform. The numbers beneath the titles indicated the number of sets completed for each posture, and a green checkmark overlay on the image indicated when data collection for a posture was complete (see ‘No Motion’ for a completed set). The red square under the images increased in size horizontally and changed colour from red, to yellow, to green as a set was completed to show the participant that data were being collected. The red circle above the images turned green when the classifier estimated that class. During data collection, the subject could also perform some postures to determine how well the classifier was estimating their movements

Each subject first collected data within the virtual environment using a PR training ‘wall’ (Fig. 2). This interface displayed the different postures and tracked the number of data sets collected for each posture. Each subject collected seven sets of data (lasting 2.5 s each) for each of the postures. The first five sets were used to train the classifier, and the final two sets were used as testing data for offline analysis. Feature extraction and LDA classification was performed in real-time, and an updated control model was built after each data set was collected to allow participants to test the classifier’s performance. Data were collected using one of two methods:

- Passive: The subject kept their arm in a single orientation, with their arm parallel to the floor and their elbow at a 90° angle, while collecting data for each of the postures. Their upper arm was held parallel to and against their body and remained still during data collection.
- Active: The subject was asked to move their arm freely around their workspace during each movement. They were encouraged to extend/flex their elbow and move their arm into a variety of orientations, such as pointing their arm upwards towards the ceiling and downwards towards the floor.

### Three-dimensional target achievement control test

**Fig. 3:**
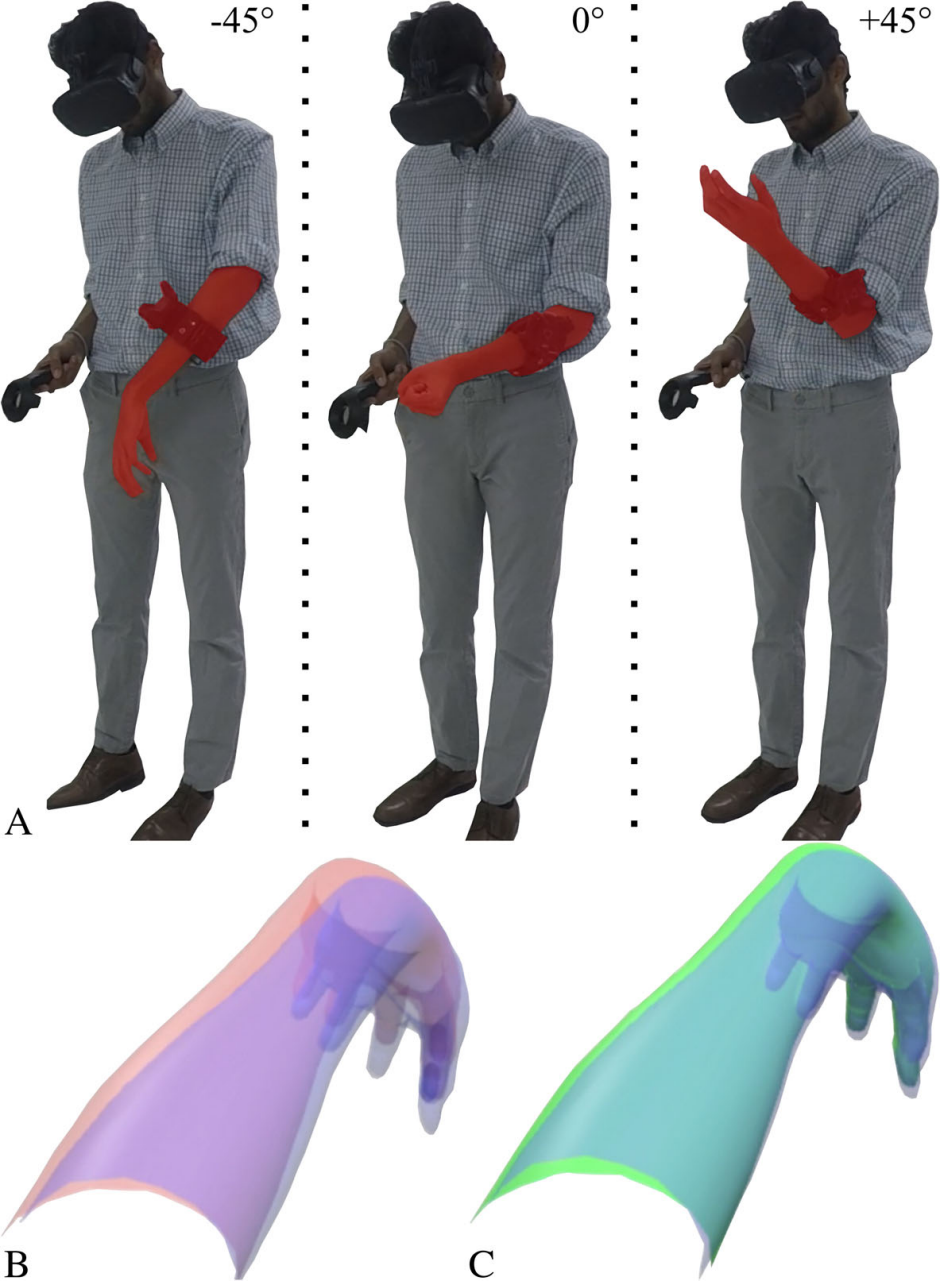
Limb orientation during study. **a** Image showing an intact limb subject with their limb in each of the three orientations: − 45°, 0°, and + 45° (left to right respectively); **b** Image showing the subject’s view during the 3D TAC test. The subject is tasked with moving their virtual arm (blue) to overlap with the target arm (red); **c** The limb turns green as an indication that the arm is correctly positioned, and the user must hold this position for two seconds

The target achievement control (TAC) test is a Fitts’-law style test that evaluates real-time myoelectric performance using a virtual 2D arm on a computer screen [31]. Here we present a 3D version of this test, in which participants must match a pseudo-randomly selected hand/wrist posture (hand open, wrist flexion, etc.) while also physically moving their limb or residual limb into one of three orientations: − 45° (arm pointing towards ground), 0° (arm parallel with ground), and + 45° (arm pointing upwards) (Fig. 3a). All required movements (except no motion) appeared randomly in each of the three orientations, twice. This resulted in 36 patterns per trial for the ITL subjects (six postures * three orientations * two repeats) and 24 patterns for the AMP population (four postures * three orientations * two repeats).

After a pseudo-random posture appeared in a pseudo-random orientation, the subject had 20 s to move their virtual limb to overlap with the target posture (Fig. 3b). After successfully achieving the posture, the virtual limb changed from blue to green. The subject then had to maintain the posture for two seconds until an audible tone was played to indicate success (Fig. 3c). If the subject failed to hold the position, an alternative tone was presented to indicate a miss.

### Pattern classification adaptation through serious gaming

#### User perspective

**Fig. 4:**
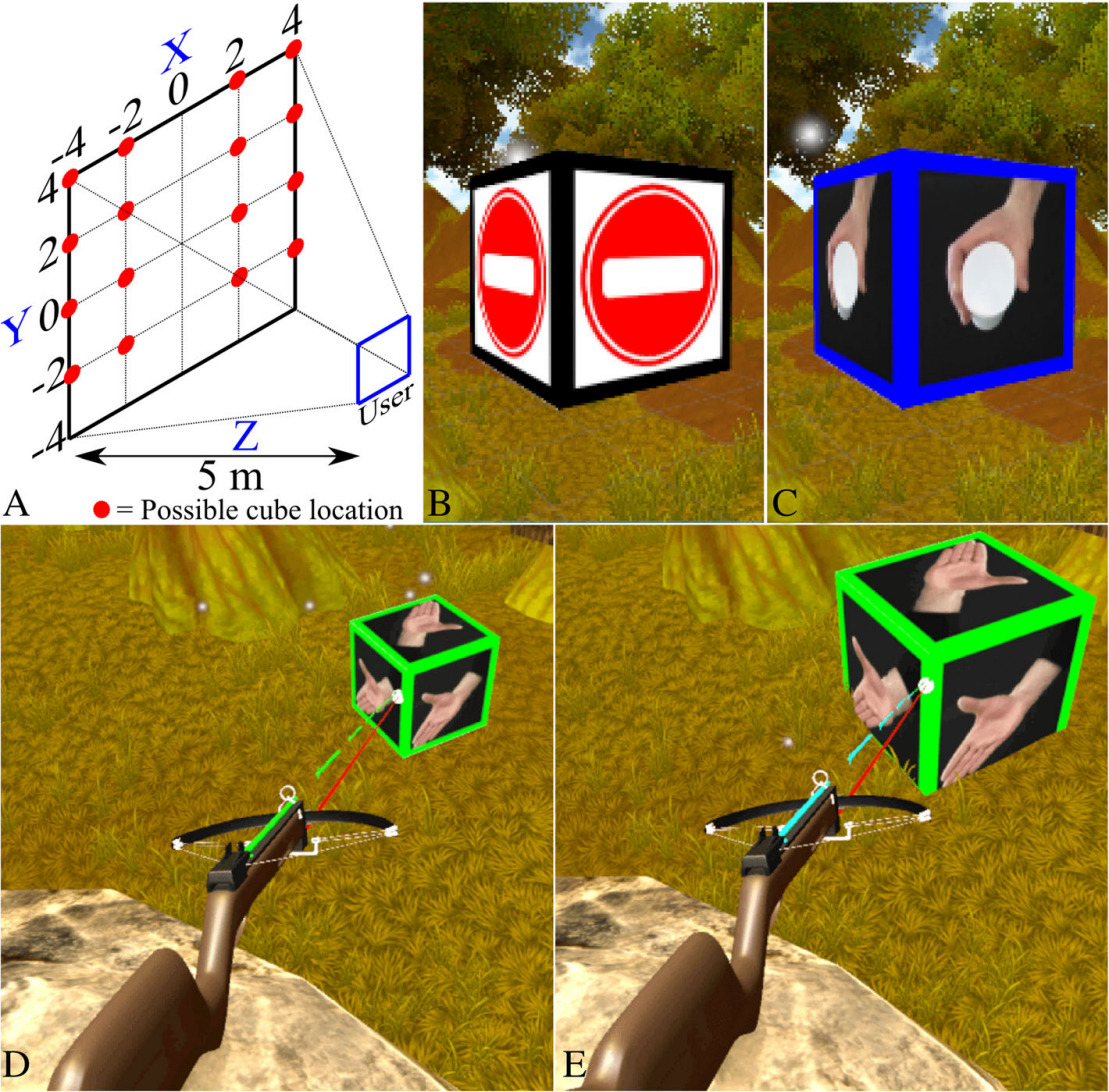
Virtual interface and environment. **a** From the user’s perspective, a cube could appear in one of four vertical positions (two on either side of the user), and four horizontal positions, some appearing above the user and some appearing below; **b** When the user was not aiming the crossbow at a cube, a non-descript image was displayed; **c** Once the user aimed the crossbow at the cube, the target posture was revealed; **d** Image shows the user beginning to interact with a cube. The target is ‘hand open’ (green) and the user is volitionally performing a hand open posture, which is resulting in green arrows being fired; **e** Image shows the moment just before the cube ‘breaks’. The cube has increased in size; however, the image shows the pattern recognition system mis-estimating, as the arrows fired are cyan (indicating a wrist flexion posture). Although arrows generated from incorrect postures did not increase the cube size, the information was added to the classifier in the adaptation group

The user was placed within a virtual forest, and, in lieu of the virtual arm used in the 3D TAC test, the trained PR system was used to control a virtual crossbow. The user faced a single direction and was presented with the task of ‘breaking’ cubes that pseudo-randomly appeared in one of 16 discrete positions in front of them (Fig. 4a). Cubes initially had a nondescript image on each face (Fig. 4b), but once the user aimed the crossbow at the cube, a target posture was revealed, together with a coloured border (e.g., a blue border for the hand-close posture, Fig. 4c). The user attempted to perform that posture, and for every estimate made by the PR system, the crossbow fired an arrow, which was colour-coded for each posture (e.g., red for no motion, blue for hand-close, etc.). The crossbow also had a laser sight that helped the user aim at the cube. Aiming the crossbow at the cube and performing the required hand/wrist posture increased the cube’s size until it eventually ‘broke’. Aiming at the cube could be achieved either through flexion/extension at the elbow (keeping the upper arm immobile against the body), or with a straight arm and flexion/extension at the shoulder. Either method was acceptable and did not change results, as in both scenarios the user had to move their forearm to the same angle to point the crossbow at the cube.

Each cube disappeared after three seconds, starting from when the user first aimed the crossbow at the cube. If the user fired only correct arrows (i.e., they were attempting the correct posture) from the moment they aimed at the cube, it took 2.5 s to break, which, together with a specific sound, indicated success. Incorrect arrows (e.g., arrows generated by a wrist flexion posture hitting a cube showing hand-close) did not increase the cube size. If the user was unable to break the cube within the time limit, the cube disappeared; these failed attempts were paired with a different sound to indicate an unsuccessful effort. The user continued to break cubes until a finish message was presented.

#### Technical elements

Each arrow represented a window of EMG data, and arrows were fired at a rate of one every 25 ms. For both groups, all postures (seven for ITL and five for AMP subjects) pseudo-randomly appeared as cubes throughout the game, except for the first three cubes, which were always ‘no movement’ to stabilise any unintentionally firing arrows at the start of the game. The serious gameplay had two modes, depending on whether the user was in the adaptation or fixed group.

For subjects in the adaptation groups (ITL and AMP), EMG information for every arrow that hit a cube during gameplay was applied to the classifier for that posture (Fig. 4d). Thus, although only correct posture estimations increased the cube size and facilitated gameplay, data from every arrow (including incorrect ones, presented visually as different coloured arrows) were applied to the classifier (Fig. 4e). The game finished once the group reached an adaptation limit, which is detailed below in the Protocol section.

For the fixed group (ITL only), the gameplay remained the same, they received the same visual and audible feedback as the adaptation group, and the game ended once they reached a time limit of five minutes, which was approximately the amount of time it took the adaptation group to reach their adaptation limit. An additional video file shows a visual description of the serious game protocol (see Additional file 1).


**Additional file 1:** Annotated video which demonstrates the serious game protocol. (MP4 7707 kb)


### Protocol

#### Familiarisation stage

To reduce learning effects, users were given six practice attempts for the initial data collection and the 3D TAC test. For each attempt, the data were reset and recollected before the 3D TAC test was performed. This familiarisation stage was performed to ensure that any improvements seen between experimental testing blocks were a result of adaptation, and not due to the subject simply getting better from experience or learning the test.

Each subject was also allowed one practice run of the crossbow game to learn how to play. As performance in the crossbow game was not evaluated, subjects did not need to become familiar with it.

#### Testing stage

The testing protocol consisted of:

1. Initial data collection using the PR training wall.
2. A TAC test to determine baseline performance, referred to as block^1^.
3. A first attempt at the crossbow serious game. The game finished once the participant collected a data ratio of 1:1 (new data to initial data) for adaptation participants, or after five minutes for the fixed group.
4. A second TAC test to determine changes in real time control after the first attempt at the crossbow game (referred to as block^2^).
5. A second attempt at the crossbow serious game. Finishing once the participant reached a data ratio of 2:1 (new data to initial data) for adaptation participants, or after five minutes for the fixed group.
6. A final TAC test to determine changes after the second attempt at the crossbow game (referred to as block^3^).

Each subject performed this protocol twice: once while performing the initial data collection passively, with their limb in the neutral position, and again while actively moving their limb during data collection. The order of passive and active data collection was randomised for each subject, with half of the subjects per population (i.e., eight subjects for ITL and two for AMP) starting with passive and the other half starting with active data collection. The TAC test and game were performed in the same way for each data collection method.

### Performance evaluation

During the TAC test, the following testing metrics were determined:

- Postures Completed: The number of pseudo-random target postures successfully achieved out of 36 (ITL) or 24 (AMP); as demonstrated in Fig. 3c. A score of 100% would mean the subject got all postures, whereas a score of 0% would mean none were achieved.
- Posture Completion Time: The average amount of time taken to achieve each pseudo-random target posture (maximum of 20 s). The time was reformatted into a proportionally scaled value and flipped, so a score of 100% would indicate that the subject got to each posture instantaneously (within zero seconds), whereas a score of 0% would mean each attempt timed out after 20 s.
- Classifier Efficacy: An altered representation of the classifier success rate. Processed window estimates ($es{t}_n^{pro}$) were calculated as a success (equal to one) if the classifier predicted (*est*_*n*_) either ‘no motion’ (*nm*) or the correct pseudo-random target posture (*t* - e.g. hand open), as seen in (1). Window estimates which predicted any other posture were deemed unsuccessful (equal to zero). The classifier efficacy was calculated as the ratio of correct to incorrect estimations across the total number of windows (*N*) and multiplied by 100 to obtain the percentage, as seen in (2). A score of 100% would mean the classifier successfully estimated the intended target posture. A score of 0% would mean that all estimates were incorrect, suggesting either poor control over the virtual limb, or a poorly trained classifier.

1 $$ {est}_n^{pro}=\left\{\begin{array}{cc}1,& \mathrm{if}\ {est}_n=t\\ {}1,& \mathrm{if}\ {est}_n= nm\\ {}0,& \mathrm{else}\end{array}\right. $$

2 $$ {eff}^{class}=\left(\frac{\sum_{n=1}^N{est}_n^{pro}}{N}\right)100 $$

- Movement Efficacy: The efficacy of the movement was evaluated by combining the classifier estimation with proportional control. Thus, this metric expressed both the altered classifier success rate (1) and the volitional speed used to move the virtual limb (*prop*_*n*_), as seen in (3). The multiplication in (3) created a similar vector to that seen in (1) whereby incorrect classifier estimations set ${ pro p}_n^{pro}$ to zero, and correct estimations (represented by estimations matching the target posture or ‘no motion’) set ${ pro p}_n^{pro}$ to the proportional value of that window (*n*) and posture. Movement efficacy (4) was calculated as the summation of the processed proportional information (3) divided by the summation of unprocessed proportional data (*prop*_*n*_ - which contains all windowed proportional information, regardless of correct estimation), and then multiplied by 100 to obtain the percentage. A correct estimation paired with an intense contraction (which was represented by a high proportional value) resulted in higher overall movement efficacy than the same correct estimation with a weak contraction/proportional value. A score of 100% would mean the subject controlled the movement correctly and smoothly, straight to the target. Likewise, a score of 0% would show the subject had poor control over the virtual limb and failed to move to the target.

3 $$ { pro p}_n^{pro}={{ pro p}_n}^{\ast }{est}_n^{pro} $$

4 $$ {eff}^{movement}=\left({\sum}_{n=1}^N\frac{{ pro p}_n^{pro}}{{ pro p}_n}\right)100 $$

These metrics were calculated for each repeat of the testing protocol for both passive and active collection methods, and changes were presented as percentages for each block. Finally, the offline accuracy of the classifier, between blocks, was calculated and averaged over all postures.

### Statistics

Statistical analysis was performed in Minitab (version 18.1). ITL and AMP populations were evaluated separately with different models for each. Statistics were performed using a mixed effects model with population subject identifier as a random factor, and block (block^1^, block^2^, block^3^), data collection type (passive or active), and (for ITL only) adaptation type (fixed or adaptation group), as fixed factors. The mixed effect model was performed separately for each testing metric (postures completed, posture completion time, classifier efficacy, and movement efficacy). The model was performed with 2nd order interactions, restricted maximum likelihood variance estimation, and a Kenward-Roger approximation test method for fixed effects. Pairwise comparisons were performed using a Bonferroni (95% confidence level) method. To further analyse statistical change between blocks, of which the mixed model is incapable of reporting, an independent analysis of variance (ANOVA) test was also performed (subject identifier as random factor, adaptation level as fixed). ITL and AMP populations were again evaluated separately, as well as separate tests for data collection type and adaptation type (ITL: adaptation and fixed, AMP: adaptation only).

## Results

### Intact limb population

**Fig. 5:**
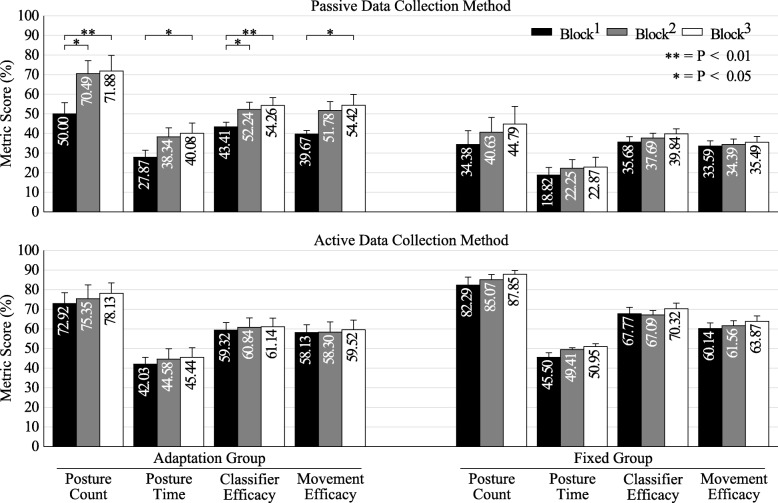
Intact limb population results. Score for each tested metric between blocks for the intact limb population’s adaptation and fixed groups. Block^1^ (black) shows the baseline result for each metric, whereas block^2^ and block^3^ (grey and white, respectively) show the change following adaptation (or lack thereof). Error bars are the standard error of the mean

Figure 5 shows the metric score across testing blocks for all tested metrics (higher is better). Table 1 shows the statistical results of the mixed model. Collection method and the 2nd order interaction between collection method and adaptation group were significant factors (*P* < 0.01) for all metrics, as was block level (*P* < 0.01 for postures complete, completion time, and movement efficacy, *P* < 0.05 for classifier efficacy). In the adaptation group with passive collection, the ANOVA showed significant improvements between block^1^ and block^2^ for the count and classifier efficacy metrics (*P* < 0.05), and significant improvements between block^1^ and block^3^ for all metrics (count, classifier: *P* < 0.01; time, movement: *P* < 0.05). None of the metrics showed significant improvements between block^2^ and block^3^ (*P* > 0.05). Separate ANOVA tests showed no significant change (*P* > 0.05) between any of the blocks for the remaining ITL group permutations (adaptation group with active collection, fixed group with passive collection, fixed group with active collection). Table 2 shows the model summary with R^2^ values between 80 and 86%, depending on the metric tested, whereas Table 3 shows the offline accuracies of the adaptation and fixed groups (higher is better). As no adaptation was performed in the fixed group, their accuracies remain unchanged between blocks.

**Table 1:** Statistical and classification results for the intact limb population: mixed model results for fixed effects

Term	F-Value				*P*-Value			
	Count	Time	Classifier	Movement	Count	Time	Classifier	Movement
Blocks	6.34	6.22	4.39	5.20	0.003††	0.003††	0.016†	0.008††
Collection (active/passive)	121.34	135.07	241.14	162.64	0.000††	0.000††	0.000††	0.000††
Groups (adaptation/fixed)	1.20	1.11	0.54	1.43	0.293	0.309	0.473	0.252
Blocks*Collection	1.96	0.64	1.90	1.54	0.148	0.529	0.157	0.221
Blocks*Groups	0.68	0.41	2.13	0.43	0.508	0.667	0.127	0.650
Collection*Groups	43.45	36.73	56.32	36.02	0.000††	0.000††	0.000††	0.000††

* indicates an interaction between two terms. †† indicates significance of *P* < 0.01, † indicates significance of *P* < 0.05, no symbol indicates no significance (*P* > 0.05)

**Table 2:** Statistical and classification results for the intact limb population: mixed model summary

Standard Error of the Estimate (S)				R^2^			
Count	Time	Classifier	Movement	Count	Time	Classifier	Movement
12.60	7.56	7.12	6.28	79.70%	81.50%	86.11%	83.00%

**Table 3:** Statistical and classification results for the intact limb population: offline classification accuracies (%)

	Passive			Active		
	Block^1^	Block^2^	Block^3^	Block^1^	Block^2^	Block^3^
Adaptation	90.65	88.40	87.83	83.38	79.82	78.40
Fixed	89.55	89.55	89.55	84.79	84.79	84.79

Offline classification accuracies across adaptation and fixed groups, and passive and active data collection methods (classification accuracies are the same for each block in the fixed demographic as no adaptation was performed and therefore the data used in offline accuracy remained unchanged)

### Amputee population

**Fig. 6:**
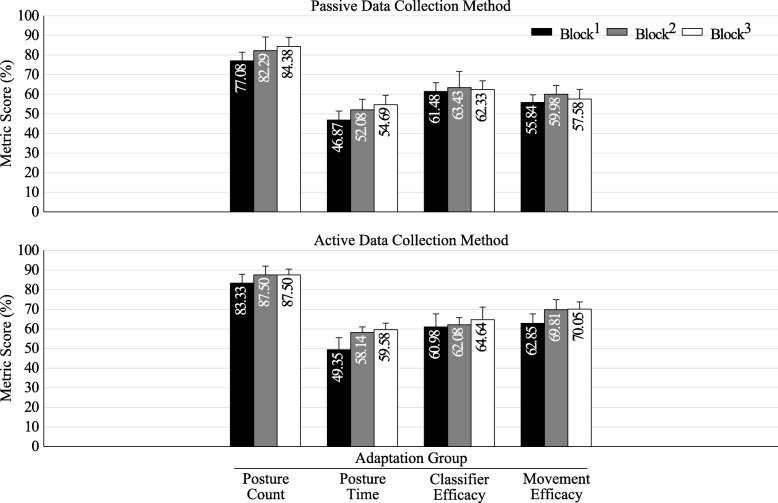
Amputee population results. Score for each tested metric between blocks for the amputee population’s adaptation group. Block^1^ (black) shows the baseline result for each metric, whereas block^2^ and block^3^ (grey and white, respectively) show the change following adaptation. Error bars are the standard error of the mean

Figure 6 shows the metric score across testing blocks for the AMP population.

Table 4 shows the statistical results of the mixed model for the AMP population. Only posture completion time for block level and the classifier efficacy metric for collection method were found to have significant effects (*P* < 0.05). Separate ANOVA tests showed no significant change (*P* > 0.05) between blocks in any of the AMP permutations (adaptation group with passive collection, adaptation group with active collection).

**Table 4:** Statistical and classification results for the amputee population: mixed model results for fixed effects

Term	F-Value				*P*-Value			
	Count	Time	Classifier	Movement	Count	Time	Classifier	Movement
Blocks	1.69	3.71	0.64	1.07	0.217	0.049†	0.542	0.369
Collection (active/passive)	3.22	2.49	4.60	3.53	0.093	0.136	0.049†	0.080
Blocks*Collection	0.12	0.14	0.42	0.50	0.892	0.872	0.665	0.618

* indicates an interaction between two terms. † indicates significance of *P* < 0.05, no symbol indicates no significance (*P* > 0.05)

Table 5 shows the model summary with lower R^2^ values between 65 and 75% depending on metric, and Table 6 shows the offline accuracies for the amputee adaptation group.

**Table 5:** Statistical and classification results for the amputee population: mixed model summary

Standard Error of the Estimate (S)				R^2^			
Count	Time	Classifier	Movement	Count	Time	Classifier	Movement
6.63	6.96	5.44	6.72	65.11%	63.39%	72.75%	75.31%

**Table 6:** Statistical and classification results for the amputee population: offline classification accuracies (%)

	Passive			Active		
	Block^1^	Block^2^	Block^3^	Block^1^	Block^2^	Block^3^
Adaptation	94.95	94.43	93.13	86.73	89.25	88.25

Offline classification accuracies across the adaptation group, and passive and active data collection methods

## Discussion

Figure 5 shows a clear advantage to training actively compared to the traditional passive data collection method. The data presented in Table 1 suggest that the method of collecting training data is a significant factor (*P* < 0.01) that impacts the resulting real-time control performance in people with intact limbs. These results support previous recommendations made in offline studies that training with the limb in a variety of orientations, or moving the limb during data collection, results in better control [13, 14]. However, these results differ from recent real-time control results evaluating changes in residual limb positions when using a regression-based control algorithm [10]. It is possible that the regression-based control is more resilient to changes in residual limb position; however, it is also possible that the inclusion of six (in the current trial) rather than four (in the prior study) postures is responsible for this difference.

Our statistical model also showed a significant interaction term (*P* < 0.01) between the data collection method (active/passive) and the adaptation group (adaptation/fixed). The mixed model was limited in that it reported significant improvements during adaptation with the passive training method but did not specify at which stage such improvements were made, and therefore ANOVA tests were applied. The significant improvements between blocks for the ITL group, with adaptation and passive collection, suggests that adaptation through the serious game could be used as an automated method to help guide collection of improved training data to provide similar performance to actively collected data supervised by an expert.

Offline analysis of results (Table 3) showed a decline in accuracy across blocks for both training data collection methods. This was expected, due to the narrowing feature space between postures as the variety of data is increased, resulting in a more complex dataset that diminished the performance of the classifier. This also agrees with prior work showing that it is possible to have better online control using PR systems with lower classification accuracies [21], and other work showing that offline performance metrics correlate only weakly with control [32].

The AMP group had differing results from the ITL participants. AMP participants performed better than the ITL group and showed a less pronounced and non-significant difference between collection methods and testing blocks (Fig. 6). This is possibly because of a reduced number of postures (five opposed to seven in the ITL group), or because changes in residual limb positioning affect amputees differently due to biomechanical differences, which may include, for example, the lack of hand and forearm weight, or differing muscle structure following amputation. It is worth noting that all participants had significant myoelectric experience, both within a controlled environment and in the home, and one of the participants had undergone targeted reinnervation surgery, which has been shown to improve myoelectric performance [33]. However, performance within the ITL group did not correlate with PR experience, as some users with no myoelectric experience performed far better than some with considerable experience. Thus, PR experience may not have affected performance in the AMP group. It is possible that performance correlates with understanding of PR concepts, rather than with experience in using PR.

The offline analysis (Table 6) also showed differing results compared to the ITL population. Although the passive data collection method also showed a decline in accuracy across blocks, albeit much less distinct, the active collection method showed an increased accuracy in block^2^, and then a slight decrease again in block^3^. These results further suggest that the amputee population has a different response to the different training methods, which may be less influential than in the ITL demographic.

It was assumed that during the crossbow adaptation game, the user was performing the correct posture according to what was shown on the cube, and that incorrect arrows fired were due to misclassification and not because the user performed the wrong posture. If the user performed a different posture to that indicated on the cube, the classifier would become saturated with incorrect data. To reduce this risk, subjects were informed that the aim of the game was accuracy and not speed, thus there was no benefit in trying to progress through the game faster, which could result in misreading the cube and performing the wrong posture.

As part of this study, each subject was given six training trials before testing to familiarise them with the protocol and to ensure that any improvements seen were due to the adaptation and not due to increasing familiarity with the test environment. To confirm this, each metric was averaged across all AMP subjects for both the last training session (the sixth repeat of the data collection/testing protocol) and the baseline result for their first testing session. A paired *t*-test showed no significant difference (*P* > 0.05) across any metric between the final training and first testing session. This suggests that by the final training session the subject’s performance had plateaued, and that the performance improvements seen were due to the adaptation and not due to more experience in the virtual environment.

Although our results show that the addition of information from many varied limb positions can improve myoelectric control, we expect that improvements would be more substantial with the inclusion of active arm movement during adaptation. Much like the baseline collection for the active collection method, moving the arm in a variety of positions during adaptation, as opposed to keeping the arm still, could have further benefits for control and is an objective of future work. Addition of such information would be computationally guided, much as performed in the crossbow game now, which would accomplish our goal of obtaining an optimally trained classifier while reducing the need for trained professionals.

Other future work includes customising and identifying each subject’s adaptation needs, so that the classifier is optimised on an individual basis. As previously mentioned, subject performance was varied, and in many ways, unpredictable. Subjects with PR experience did not always perform well, and some novice users performed exceptionally well.

## Conclusion

This study shows that an actively trained classifier performs better than one trained in a single, neutral orientation, and that adaptation within a virtual guided serious game environment can improve real-time performance of myoelectric controllers; however, this was only significant in the ITL group in this study.

These results concur with previous work showing that data collection during active movement and in a variety of arm orientations and positions can significantly improve classification and control of a myoelectric prosthesis. Furthermore, we have shown that collecting data, with minimal instruction, in the traditional neutral orientation can be significantly improved through a computationally guided tool using virtual reality and serious gaming.

Although the virtual training tool is not designed to replace professional interaction or clinical care, it can assist with training or retraining a PR system in a home or semi-controlled environment. Future work will include reducing the technological requirements by porting this tool to mobile devices and using less computationally heavy and expensive systems.

## Abbreviations

AMP: Amputee
DOF: Degree(s) of freedom
EMG: Electromyographic/electromyography
ITL: Intact limb
LDA: Linear discriminant analysis
PR: Pattern recognition
TAC: Target achievement control
VR: Virtual reality
